# The Germ Theory of Disease

**Published:** 1880-04

**Authors:** John A. Henning

**Affiliations:** Redkey, Ind.


### Selections.

### THE GERM THEORY OF DISEASE.

By JOHN A. HENNING, M.D., Redkey, Ind.

The germ theory of disease is not by any means a new question. Linneus, the father of botany, over a century ago, first propounded this theory, and gave it considerable attention; but he was alone in the study, and made but little progress, though his researches were of permanent value. But as time advanced other scientific men entered the field, who made some valuable discoveries and observations, and reported their results.

Prof. Obermier, of Berlin, discovered minute fungoid growths, called the spirillium, in the blood of patients suffering with relapsing fever.

Prof. Stricker, of Germany, says: "Of all the modifications which the blood undergoes, the least understood, and at the same time the most important, is unquestionably that which is due to the admixture with low organisms.

Prof. Klein, of London, discovered a kind of bacterium in the peritoneal exudation in pigs affected with pneumo-enteritis.

Dr. Sensino, an Italian physician, " found that in nine necroscopies made, seven of the cadavers contained the parasite sclerostoma, firmly attached to the mucous membrane of the duodenum and jejunum. After eroding this membrane they suck out the blood, and if there are many of them present, invariably produce intense anemia. In a woman dying of anemia at the hospital of Florence, the intestines were found to be very thin, pallid and almost transparent. Hundreds of these parasites, the sclerostoma, were in the jejunum and ileum. They were dead and attached to the mucous membrane, which presented corresponding grayish echymoses. No other lesions were found sufficient to account for the profound anemia."

Dr. Neftel, of New York, in 1868, says: "My experiments so far lead me to the conclusion that the lower vegetable organisms can continue to live and multiply in the tissues of living animals, and that they can enter into the general circulation, either through the intestinal canal, or through the respiratory organs, or by means of hypodermic injections. What their ultimate fate is in the animal organism, and what their importance in producing disease, further investigations will have to show."

Dr. Wartabet, of Edinburgh, says: "The plague is one of those zymotic diseases whose origin is still unknown, though recent observers incline to the theory that they are produced by germs, as minute organisms, generated under favorable conditions, and which enter the blood and give rise to well defined morbid phenomena."

Dr. Lawson has found the fluid of pyrosis to contain sarcinæ, torulæ, huge clusters of leptothrix, and myriads of vibrios and bacteria, which, by their presence, keep up the irritation, causing pyrosis and mucous dyspepsia.

Prof. Scudder says; "One of the finest hunting grounds for microscopic germs is a dirty mouth; and I have counted forty-three varieties and species in the dirt of the skin."

Prof. Richardson, of Philadelphia, in 1868, experimented by drinking four ounces of water supposed to contain twenty-seven billions of bacteria; in a few hours afterward he found bacteria in his blood, without any perceivable harm. But he further says "the bacterial spores and rods are only the analogues of the seeds and roots of larger plants, and by no means entire organisms."

"Lord Somerville gave an interesting account of the effect of a punishment which formerly existed in Holland. The ancient laws of the country ordained men to be kept on bread alone, unmixed with *salt*, as the severest punishment that could be inflicted upon them in that moist climate. The effect was horrible. Those wretched criminals are said to have been devoured by worms engendered in their own stomachs."

Prof. Stille, when speaking of comparative pathology of cholera, yellow fever, and malignant fever, says about the cause, that it is "evidently something received from without and swallowed; one opinion is, that it consists of certain microscopic fungi, or their germs."

Dr. W. Robberts, of Manchester, England, says in his annual address before the British Medical Association in 1877 that "the germ theory of disease is now established upon a firm experimental basis, and if fairly grasped in capable hands, will very soon give us most important aid in the struggle with disease."

Other most excellent testimony from scientific research might be produced, but the above is sufficient to answer our purpose, in establishing the question upon a firm foundation.

It is said by scientists that there are over twenty-five hundred species and varieties of those (invisible) germs, by which we are surrounded, and which permeate our bodies, inhabiting the various organs and tissues. Upon this basis the germ theory of disease proposes to account for the original causes and phenomena, of all the contagious diseases. These spores, or fungi, have the power, in a good nidus and under favorable circumstances, of multiplying with great rapidity. When the system is in a normal condition, these germs are harmless; but the slightest deviation from health gives them an excellent nidus, and under favorable circumstances they reproduce themselves rapidly. The soil, locality, climate, warmth and moisture, or dryness, aid materially in their production.

Germ signifies the entire process in which a new being originates, either vegetable or animal. The germs are so small that millions may be contained in one sixty-fourth of an inch. A parasite is a living organism, springing from the germ, but living upon another organism. Fungi are morbid growths originally from the germ. With this explanation our propositions may be somewhat better comprehended, and we will proceed to particularize as to some of those parasites which cause certain diseases.

Bacteria and micrococci are the parasites causing dyphtheria; saracenia cause variola; fungi, measles; amebæ, with their associates forming colonies, many diseases of the respiratory organ, such as some forms of pneumonia, asthma, catarrh, laryngitis, and bronchitis; sarcinæ ventriculi, many forms of dyspepsia; vibrios, general nervous depression, but particularly typhoid fever; the oidium albicans, and its associates, pseudo-membranous croup, mycelium, whooping cough; trichina spiralis, trichinosis; bacteria and oidium albicans, erysipelas.

These parasites very generally unite and form colonies, and certain species have a particular affinity for certain parts, or organs of the system, producing certain diseases. They live in the healthy system with harm, but it furnishes an unfavorable soil.

We are now prepared to ask, what is life in health, and what in disease, especially in relation to germs? The protoplasm of the cryptogamic family in which mankind is more or less surrounded, is continually working, but so long as the vital forces are perfect, these forms are powerless, or lie dormant in the system. But in depression, either general or local, these germs, or seeds, may immediately, by cell-multiplication, produce certain diseases. The living cells of our bodies must not be confounded with germs. The parasite is a special being possessing individuality, is an organism living upon an organism. Some of these germs live exclusively in cold weather, others in warm weather.

I am aware this germ theory of disease is yet in its infancy, but admitting it to be correct, it seems as though we will have to study our materia medica anew. In combatting terrible diseases, we must understand the primary cause. We must know what remedy to give, and why we give it. Prof. Lister has shaken the medical world from center to circumference, by his *antiseptic treatment.* His method is rather preventive than curative. Diluted carbolic acid applied to wounds prevents the bacteria and other parasites from forming, and thus wounds heal by first intention. Otherwise a low, parasitic grade of inflammation and suppuration would be the result. Prof. Whitford, of Chicago, says that terebinthina will utterly destroy the trichina spiralis, when given internally and applied externally.

The reason why hydrochloric acid, turpentine, carbolic acid, quinia, and nitro-muriatic acid are excellent remedies in typhoid fever, is because they destroy the *vibrinous*. Acetic acid will destroy the mycelium, which is recognized in diphtheria andpseudo-membranous croup. Borate of soda and salicylic acid are destructive of a species of oidium albicans, which produces many cases of leucorrhea.

In dyspepsia, due to sarcinæ ventriculi, sulphite of soda will soon effect a cure.

Another large field for further scientific investigation is in reference to the effects of fermentation of food in the stomach. We have sufficient testimony to believe that *germs* are intermixed in many kinds of food we eat, and when fermentation takes place in the stomach, the germs find an excellent nidus.

Dr. Crowther, of London, recently said: "There can be no doubt that we have in the hyposulphite of soda one of the most valuable remedies for a large class of fungoid diseases, also many varieties of zymotic affections. It is also a potent remedy in certain intractable acute and chronic ulcers, whose origin and continuance seem due to some local irritant of a fungoid or bacterial nature." This is doubtless correct, and I have used the sulphite of soda in the same class of diseases with most excellent success. These germs cannot be eliminated from the system alive; they must be destroyed, when they will pass out of the system, through the various channels, the same as worn out tissue.

The ameba is a winter parasite, found in a number of respiratory diseases, and we get excellent results from the use of muriate and carbonate of ammonia, which destroy the parasite.

A very large per cent. of skin diseases are caused by either a vegetable or animal parasite. Some of these are confined to the skin, others are so subtle that, through the fluids and lymphatics, they quickly migrate to internal organs. This is particularly noticed in measles and scarlatina.

Thus it will be noticed that progress is being made in the germ theory. Valuable thoughts and discoveries are made daily in this direction. The duty of every physician in the land is to procure a good microscope, and to use it at every leisure moment he may have, especially during the prevalence of an endemic or epidemic disease. Thus each will be enabled to make his own discoveries, and present his mite to the profession for public inspection.

